# Effect of cytochrome P450 inhibition on toxicity of diclofenac in chickens: Unravelling toxicity in *Gyps* vultures

**DOI:** 10.4102/ojvr.v89i1.1978

**Published:** 2022-06-14

**Authors:** Sara Locke, Vinny Naidoo, Ibrahim Hassan, Neil Duncan

**Affiliations:** 1 Department of Paraclinical Sciences, Faculty of Veterinary Science, University of Pretoria, Pretoria, South Africa

**Keywords:** diclofenac, toxicity, vulture, cytochrome P450, pharmacokinetics, chicken

## Abstract

Diclofenac was responsible for the decimation of *Gyps* vulture species on the Indian subcontinent during the 1980s and 1990s. *Gyps* vultures are extremely sensitive (the lethal dose 50 [LD_50__]_ ~ 0.1 mg/kg – 0.2 mg/kg), with toxicity appearing to be linked to metabolic deficiency, demonstrated by the long T_1/2_ (~12 h – 17 h). This is in striking comparison to the domestic chicken (*Gallus gallus domesticus*), in which the LD_50_ is ~10 mg/kg and the T_1/2_ is ~1 h. The phase 1 cytochrome P450 (CYP) 2C subfamily has been cited as a possible reason for metabolic deficiency. The aim of this study was to determine if CYP2C9 homolog pharmacogenomic differences amongst avian species is driving diclofenac toxicity in *Gyps* vultures. We exposed each of 10 CYP-inhibited test group chickens to a unique dose of diclofenac (as per the Organisation for Economic Co-operation and Development [OECD] toxicity testing guidelines) and compared the toxicity and pharmacokinetic results to control group birds that received no CYP inhibitor. Although no differences were noted in the LD_50_ values for each group (11.92 mg/kg in the CYP-inhibited test group and 11.58 mg/kg in the control group), the pharmacokinetic profile of the test group was suggestive of partial inhibition of CYP metabolism. Evaluation of the metabolite peaks produced also suggested partial metabolic inhibition in test group birds, as they produced lower amounts of metabolites for one of the three peaks demonstrated and had higher diclofenac exposure. This pilot study supports the hypothesis that CYP metabolism is varied amongst bird species and may explain the higher resilience to diclofenac in the chicken versus vultures.

## Introduction

The use of chemicals to exert a biological effect has long been understood and utilised by humans. Today, it can be little disputed that the use of modern chemical compounds in the human and veterinary sector has had profound influence on health and disease management, agriculture and the economy at large. The impact of pharmaceuticals on the environment has, on several occasions, been the price we have paid for these advancements. A historical yet infamous example is the pesticide dichlorodiphenyltrichloroethane (DDT). Whilst controlling crop-destroying insects in the United States (US) and assisting with mitigating the spread of diseases like malaria in Africa, DDT had devastating effects on other wildlife species, most notably raptorial birds of prey such as the peregrine falcon (*Falco peregrinus*) and the bald eagle (*Haliaeetus leucocephalus*) (Köhler & Triebskorn 2013; Rattner 2009).

In the veterinary context, animal pharmaceuticals have not been exempted from their share of impact on the environment, and one of the biggest impacts of any veterinary drug has been that caused by the non-steroidal anti-inflammatory drug (NSAID) diclofenac. Used for the relief of pain and inflammation in domestic ruminants on the Indian subcontinent, diclofenac decimated populations of *Gyps* vulture species in just 20 years, the so-called ‘Asian Vulture Crisis’ (Prakash et al. 2007). These birds seemed to be susceptible to the drug at a low LD_50_ of 0.1 mg/kg – 0.2 mg/kg (Swan et al. 2006), with exposure resulting from a single engorgement at a carcass.

In comparison, the LD_50_ in the domestic chicken (*Gallus gallus domesticus*) was 9.8 mg/kg (Naidoo et al. 2007) and as high as 616 mg/kg in the pigeon (*Columba livia domestica*) (Hassan et al. 2018). The difference in LD_50_ also appeared to be correlated with the pharmacokinetic parameter T_1/2_ or half-life of elimination, suggesting deficient metabolism as a cause of increased sensitivity. The T_1/2_ is long in *Gyps* vultures (> 7 h) and short in the chicken and pigeon (< 7 h).

A plausible explanation for these striking interspecies differences could lie in the avian clade evolutionary history. Whilst birds appear to have evolved from only three major groups (the Palaeognathae, the Galloanserae and the Neoaves) (Watanabe et al. 2013), they diverged greatly over the millennia into the thousands of species present today, and their ability to metabolise xenobiotic compounds most likely diverged concurrently in accordance with difference of diet and habitat (Hutchinson et al. 2014; Thomas 2007; Watanabe et al. 2013).

Xenobiotic metabolism is divided into phase 1 and phase 2 reactions. These enzymatic processes serve, for the most part, to reduce biological activity of a compound and facilitate its excretion by the body (Brunton, Hilal-Dandan & Knollmann 2018). Of the groups of enzymes responsible for these phases, the cytochrome P450 (CYP) superfamily of phase 1 reactions is by far the largest and most diverse across animal species (Brunton et al. 2018; Hutchinson et al. 2014; Konstandi, Johnson & Lang 2014).

As CYP enzymes are renowned for interspecies pharmacogenomic variation and appear to be active in *Gyps* vultures, they have been cited as the most likely cause of diclofenac metabolic deficiency in these birds (Hassan et al. 2018; Fourie et al. 2015; Naidoo et al. 2010, 2018). In humans, diclofenac is largely metabolised by phase 1 CYP2C9 activity (Brunton et al. 2018; Daly et al. 2007; Leemann, Transon & Dayer 1993). Homologous CYP2C9 enzymes have also been found to be present in the domestic chicken (Kawalek et al. 2006; Shafi et al. 2015; Watanabe et al. 2013). In the case of *Gyps* vultures, it is hypothesised that their sensitivity to diclofenac may be as a result of a homologous CYP2C9 deficiency (Hutchinson et al. 2014; Naidoo et al. 2010, 2011).

Whilst there is speculation that an underlying CYP enzyme deficiency is responsible for the toxic effect of the drug because of zero order metabolism, the absence of the enzyme is not easy to prove as absence can be a true or a false result. An alternate manner of proving the role of a specific enzyme in toxicity would be to take a species where the metabolism is known to be present and inhibit it. If a deficiency defines toxicity, inhibiting the naturally present system should create the same level of toxicity as for the species deficient in it. A useful feature of CYP enzymes is that they are subject to inhibition of activity by chemical means. This feature can be exploited; inhibition of a specific enzyme by one drug should alter the pharmacokinetics of another drug that is a substrate for the same enzyme. The primary aim of this study was to ascertain the impact of CYP2C9 inhibition in the chicken in terms of pharmacokinetic profiles, LD_50_ and metabolites formed.

## Materials and method

### Animal housing and care

Thirty day-old Ross broiler chickens (*Gallus gallus domesticus*) weighing an average of 45.13 g were purchased for the study. The birds were housed at the University of Pretoria Biomedical Research Facility (UPBRC), Faculty of Veterinary Science. The birds were housed on wood shavings on a light-dark cycle of 10 h/14 h, with reducing temperatures from 30.6 °C to 19 °C and humidity fluctuating between 20% and 60%. The birds had ad lib access to food (poultry standard ration, according to age) and municipal potable water, and they were individually identified with unique wing-tags. At five weeks of age, the birds were allocated to the two treatment groups to achieve a group size of ten. The study protocol was approved by the University of Pretoria Animal Ethics Committee (AEC) and the Faculty of Veterinary Science Research Ethics Committee (REC).

### Study design

The study design followed the OECD guidelines for acute avian oral toxicity testing (OECD 2016), with the test group on diclofenac sodium (Panamor 75^®^, Aspen Pharmacare Holdings) and fluconazole (Diflucan^®^, Pfizer) and the control group on diclofenac sodium alone (*n* = 10). Fluconazole was used as the CYP2C9 inhibitor (Brunton et al. 2018) at 15 mg/kg (Carpenter & Marion 2018), intra-peritoneally (i/p) for three days prior to the start of diclofenac dosing in the test group. The test and control groups were subsequently dosed with diclofenac sodium. Each bird within a group was dosed with a unique dose, spaced around the working LD_50_ of 9.8 mg/kg (Naidoo et al. 2007) on a log scale, by intravenous (IV) injection, according to the OECD maximum likelihood estimation method (OECD 2016). The specific doses were 3.36 mg/kg, 4.26 mg/kg, 5.40 mg/kg, 6.86 mg/kg, 8.70 mg/kg, 11.04 mg/kg, 14.00 mg/kg, 17.77 mg/kg, 22.55 mg/kg and 28.61 mg/kg. Following the administration of diclofenac, the birds were monitored for a 15-day period. Birds still alive after 15 days were terminated by sedation and carbon dioxide asphyxiation.

### Sampling

Blood samples were drawn from the wing veins of the birds using 21 gage (G) needles attached to preheparinised 3 mL syringes at 0.0 h, 0.25 h, 0.5 h, 1.0 h and 2.0 h after dosing. Samples were transferred into prelabelled plasma tubes and centrifuged within 1 h of sampling at 1660 × g and 25 °C for 15 min. Samples were split for uric acid and diclofenac analysis and were then stored at –80 °C. Scheduled and unscheduled deaths were submitted to the University of Pretoria Paraclinical Department, section of veterinary pathology, for full gross and histological evaluation. Where needed, samples were collected in 10% buffered formalin, sectioned and stained via haematoxylin & eosin (H&E) technique and subjected to histopathologic examination.

### Drug analysis

Samples were analysed at the University of Pretoria, Veterinary, Paraclinical Department, toxicology and pharmacology laboratory. Diclofenac sodium, 4′-hydroxydiclofenac, 5′-hydroxydiclofenac and fluconazole analytical standards were purchased from Sigma Aldrich (Merck, South Africa). In short, 200 μL of plasma was placed in a 2-mL tube, followed sequentially by 400 μL of diethyl ether and 400 μL of potassium dihydrogen phosphate (0.3 M and pH 3.5). After mixing, tubes were then centrifuged for 20 min at 5878 × g and 4 °C and placed in an ice bath of methanol and CO_2_ for 3 min, which allowed the organic layer to be separated from the aqueous phase. The separated organic phase was dried under nitrogen flow at 50 °C until dry. For the high-performance liquid chromatography (HPLC) analysis, 400 μL of the mobile phase, consisting of a 42.5:57.5 ratio of sodium dihydrogen phosphate (0.05 M and pH 4.86–4.88): acetonitrile, was added to dissolve the dried sample residue.

A Beckman System Gold analyser with a 32 Karat™ software package, a diode array detector (DAD) 168, an autosampler module 508 and a programmable solvent module 126 (Beckman Instruments, Fullerton, California, US) was used for the HPLC analysis with a 250 mm × 4.6 mm, 5 μ base deactivated silica (BDS) HYPERSIL Phenyl column. A mobile phase of ratio 42.5:57.5 sodium dihydrogen phosphate (0.05 M and pH 4.86–4.88) : acetonitrile was used for both diclofenac and metabolite detection. For analysis, 30 μL was injected at 1 μL/min, and detection at 275 nm. The gradient method of analysis, with a total runtime of 8 min, was used to gradually decrease the ratio of sodium dihydrogen phosphate and acetonitrile from its starting ratio to 20:80 over a period of 3 min. The mean retention time for diclofenac and the 4′- and 5′-hydroxydiclofenac metabolite combination was 4.51 min and 3.45 min, respectively. The standard calibration curve showed an *r*² value > 0.99. The limit of detection (LOD) and limit of quantification (LOQ) were 0.195 μg/mL and 0.396 μg/mL, respectively, for diclofenac, with a range of 0.718 μg – 25.000 μg. For the 4′- and 5′-hydroxydiclofenac metabolites, the LOD and LOQ were both 0.095 μg/mL, with a range of 0.095 μg – 3.125 μg.

### Diclofenac pharmacokinetic and statistical analysis

Concentration-time data generated following HPLC analysis was dose equalised to 1 mg/kg and evaluated by non-compartmental modelling (Kinetica 5.1, Thermo Scientific). The pharmacokinetic parameters maximum plasma concentration (C_max_) and time to maximum plasma concentration (T_max_) were read off the curve. Area under the plasma concentration versus time curve to the last quantifiable time point (AUC_last_) and area under the moment curve (AUMC) were calculated using the linear trapezoidal rule as follows:

$$•\;AUC_{last}=\sum_{i=1}^{n}0.5(C_{i}+C_{i+1})\Delta \;t$$ [Eqn 1]

And

$$•\;AUMC=\sum_{i=1}^{n}0.5(C_{i}.t_{i}+C_{i+1}.t_{i+1})\Delta \;t,$$ [Eqn 2]

where *C* is the diclofenac concentration and *t* is the time.

Area under the plasma curve extrapolated to infinity was calculated as:

$$•\;AUC_{inf}=AUC_{last}+AUC_{extra},$$ [Eqn 3]

where $AUC_{extra}=\frac{C_{last}}{\beta },{\text{C}}_{\text{last}}$

is the last measured concentration and β is the terminal elimination portion of the curve gradient, on a natural logarithmic scale.

The half-life of elimination (T_1/2_) was calculated as:

$$•\;T_{\frac{1}{2}}=\frac{Ln\;2}{\beta }.$$ [Eqn 4]

Whole plasma clearance (Cl) was calculated as:

$$•\;Cl=\frac{Dose}{AUC_{last}}.$$ [Eqn 5]

Volume of distribution (Vd) was calculated as:

$$•\;Vd=\frac{Dose}{CP_{0}},$$ [Eqn 6]

where CP_0_ was the calculated concentration at time 0.

The mean residence time (MRT) was calculated as:

$$•\text{ MRT}=\frac{Dose\;Cp_{0}}{\beta }.$$ [Eqn 7]

### Uric acid analysis

Plasma samples were evaluated by the University of Pretoria, Companion Animal Clinical Studies Department, clinical pathology laboratory. Samples were analysed on a Cobas Integra 400 plus analyser (Roche Diagnostics, Mannheim, Germany), using the uricase enzyme colorimetric test, by measuring the absorbance of the red colour intensity of quinone-diimine dye formed at the end of the reaction at 552 nm. The reference range used for normal chicken uric acid concentration was based on Ross et al. (1978) and Wilson and Miles (1988). Uric acid AUC_last_ values were calculated using the linear trapezoidal rule. Baseline corrections per scheduled time points (difference between 0 h) were also calculated.

### Data analysis

The LD_50_ was determined using the maximum likelihood estimator (MLE) probit model (OECD 2016) using a chi-square goodness-of-fit model. Microsoft^®^ Excel workbook SEquential DEsign Calculator (SEDEC) was used for these calculations. For the pharmacokinetic data, results were presented as mean and standard deviation. Parameters were also subjected to univariate analysis of variance (ANOVA) analysis. Uric acid concentrations, uric acid AUC_last_ values and baseline corrections were compared using a Student’s *t*-test. For the metabolite analysis, three distinct peaks, suspected to be metabolites (not visible in blank plasma), were seen on HPLC analysis. Because of inability to separate standard 4′-hydroxydiclofenac and 5′-hydroxydiclofenac peaks on calibration and extreme variation in profiles produced between birds, metabolite peaks were analysed and compared by first calculating the dose equalised AUC_last_ values for each peak, based on the peak height readings (response) over time. These values were compared to the diclofenac peak dose equalised AUC_last_ values, per treatment group and mortality status. The metabolite AUC_last_ results were further subject to binary logistic regression to check for relationship and significance thereof between the extent of exposure and mortality. The treatment groups were compared by subjecting the means of the ratio of diclofenac : metabolite AUC_last_ peak to a Student’s *t*-test. All statistical analysis was in Statistical Package for Social Sciences (SPSS) version 1.0.0.95 statistical software (IBM, New York, NY, US).

### Ethical considerations

This study was approved by the Animal Ethics Committee (AEC) of the University of Pretoria (number: V078-18).

## Results

### Clinical signs and mortalities

In the test group, the two birds receiving the highest dose died immediately after diclofenac dosing, from what we believe to be acute cardiovascular collapse. Two birds (dosed at 14.00 mg/kg and 6.86 mg/kg body weight [BW]) died within approximately 48 h of dosing. These birds showed expected typical signs of diclofenac toxicity: lethargy and depression, anorexia and unwillingness to move. The remaining birds showed no obvious signs of intoxication. The LD_50_ was calculated at 11.92 mg/kg BW, with the 95% confidence intervals of 3.87 mg/kg and 61.87 mg/kg and a probability of 0.56. In the control group birds, one bird (dosed at 5.40 mg/kg BW) died 15 min after dosing, also from what was likely cardiovascular collapse. A second bird (dosed at 28.61 mg/kg BW) died approximately 7 h after dosing with minimal symptoms, whilst a third (dosed at 17.70 mg/kg BW) died at approximately 28 h with expected typical signs. A fourth bird (dosed at 22.55 mg/kg BW) was euthanised at approximately 56 h after dosing. The remaining birds showed no obvious signs of intoxication. The LD_50_ for diclofenac in control birds was calculated at 11.52 mg/kg BW, with the 95% confidence intervals of 3.31 mg/kg and 78.62 mg/kg and a probability of 0.78.

### Pathology

**FIGURE 1:**
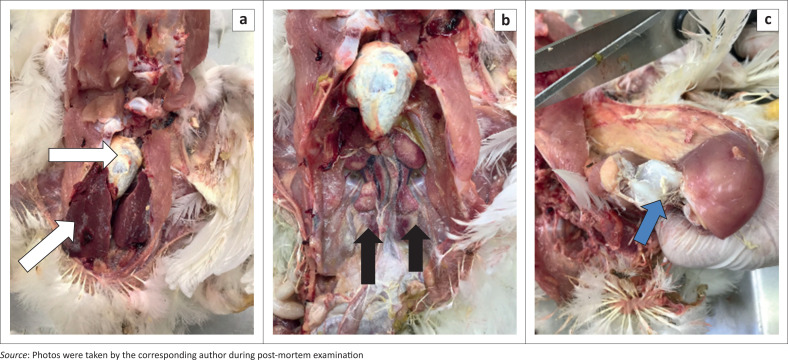
Gross pathology findings in a test group bird that succumbed approximately 48 h following intravenous administration of diclofenac. (a) Visceral gout with uric acid accumulation on the serosal surface of the liver and pericardium (white arrows), (b) nephrosis of the kidneys evident as pale and yellowish (black arrow) and (c) uric acid accumulation in the tibeo-tarsal joint indicative of articular gout (blue arrow).

The birds that died within 15 min of treatment, irrespective of the group, showed no gross or histopathological lesions. For the other birds that died within 56 h, irrespective of the group, there was a mild to marked urate deposition on all serosal surfaces and in varying degrees in the pericardium, epicardium and air sacs. Urate crystals were also seen on the liver and spleen and in the joints and tendon sheaths of the legs (Figure 1). Histopathologically, there was mild to marked widespread cell injury and necrosis of the renal tubular epithelium, with dilatation of the damaged tubules and marked deposition of globule urates within the lumens of the damaged tubules. Cell changes ranged from increased eosinophilia to cell membrane disruption and sloughing into the lumen, with spicules of uric acid visible within the eosinophilic masses. In some birds there were many urate tophi showing giant cell aggregates at the periphery.

### Diclofenac pharmacokinetic analysis

**FIGURE 2:**
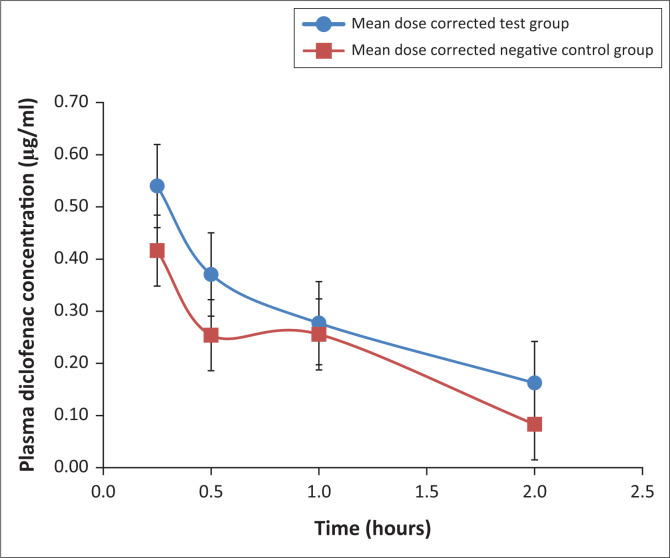
Mean diclofenac plasma concentration time curves by treatment group, dose corrected to 1 mg/kg.

The pharmacokinetic parameters per treatment group and mortality status are presented in Table 1, and the mean plasma concentrations versus time profiles are presented in Figure 2. The majority of the birds showed the expected i/v curve of linear depletion from a maximum concentration at the first point of sampling. One bird from the test group (dosed at 8.70 mg/kg BW) had a 2 h outlier reading which was impossibly high, with the result that we omitted the data point from the pharmacokinetic (PK) and statistical calculations. Another bird from the test group (dosed at 6.86 mg/kg BW) had a very high 15 min reading. The latter was consequently omitted entirely from the ANOVA calculations but included in the descriptive statistics in Table 1. Three birds, two from the control group and one from the test group, showed absorptive components, likely because of accidental subcutaneous dosing. The mean C_max_ and T_max_ values were 0.51 mg/mL and 0.38 h, respectively.

**TABLE 1:** Pharmacokinetic parameters for diclofenac in test and control birds following intravenous diclofenac exposure. Presented as geometric means (GM) on dose-corrected parameters, with the standard error of the mean (SEM).

Parameter	Test group (*n* = 8)				Control group (*n* = 9)			
	Alive		Dead		Alive		Dead	
	*GM*	SEM	*GM*	SEM	*GM*	SEM	*GM*	SEM
T_max_ (h)	0.35	0.29	0.25	0.00	0.40	0.16	0.40	0.25
C_max_ (μg/mL)	0.48	0.15	1.24	1.24	0.52	0.08	0.24	0.09
AUC_last_ (μg/mL*h)	0.42	0.10	0.87	0.73	0.46	0.07	0.31	0.07
AUC_inf_ (μg/mL*h)	0.55	0.17	1.13	1.16	0.57	0.16	0.76	0.24
Lz (1/h)	0.82	0.27	0.72	0.31	0.88	0.16	0.34	0.48
AUMC_last_ (μg/mL*(h)^2^)	0.34	0.08	0.63	0.64	0.35	0.08	0.27	0.06
T_1/2_ (h)	0.85	0.25	0.96	0.41	0.79	0.19	2.03	1.92
MRT (h)	1.34	0.31	1.33	0.44	1.17	0.27	3.14	2.78
Cl (L/h*Kg)	1.83	0.6	0.88	0.91	1.74	0.32	1.32	0.60
V_z_ (L/Kg)	2.24	0.73	1.23	0.62	1.98	0.26	3.88	2.13
V_ss_ (L/Kg)	2.44	0.69	1.17	0.71	2.04	0.20	4.16	2.07

T_max_, Time to maximum plasma concentration; C_max_, Maximum plasma concentration; AUC_last_, Area under the curve to the last measured (quantifiable) time point; AUC_inf_, Area under the curve extrapolated to infinity; L_z_, Terminal elimination phase rate constant; AUCM_last_, Area under the moment curve to last measured (quantifiable) time point; T_1/2_, Half-life of elimination or terminal half-life; MRT, Mean residence time; Cl, Clearance; V_z_, Volume of distribution during the terminal phase; V_ss_, Volume of distribution during steady state; GM, geometric means; SEM, standard error of the mean.

Analysis of variance showed no significant difference between groups for C_max_, AUC_last_ or T_1/2_, although the values for C_max_ and AUC_last_ were, on average, higher for the test group, driven largely by the values from the birds which died (Table 1 and Figure 2). Whilst the mean T_1/2_ value for the test group was lower than that of the control group (also driven largely by values from the birds that died), this must be interpreted with caution as we were unable to get values for the two highest-dosed birds in the test group because of early mortality. As evidence of the impact this may have had, the corresponding birds in the control group receiving the highest doses had long T_1/2_ values (7.01 h and 2.75 h).

### Metabolite analysis

When evaluating metabolite peak AUC_last_ values, there was no statistically significant relationship between the extent of metabolite exposure and mortality nor between dose and metabolite production. There was also no statistically significant difference in exposure between treatment groups. When comparing the two treatment groups (both dead and alive birds), the test birds produced higher AUC_last_ metabolite values compared to the control birds (Table 2). The exception to this was peak 3 in the birds that died, which was notably lower in the test birds (value in bold in Table 2). The mean dose-corrected diclofenac peak AUC_last_ reading was also considerably higher in the dead birds (value in bold in Table 2). A different picture was present when comparing birds that died with those that survived across both treatment groups (Table 2); the birds that died produced lower AUC_last_ metabolite values for all three metabolite peaks across both groups. The exception was peak 3, where the mean AUC_last_ values were almost identical between dead and alive birds within the control group.

**TABLE 2:** Mean dose-corrected area under the curve to the last measured (quantifiable) time point values for each metabolite peak and diclofenac per treatment group.

Mortality	Peak	Test group			Control group		
		Mean AUC_last_† (m Au)	s.d.	%CV	Mean AUC_last_† (m Au)	s.d.	%CV
Alive	1	676.00	432.09	63.92	383.81	248.19	64.67
	2	256.68	97.44	37.96	180.95	109.04	60.26
	3	470.09	630.59	134.14	243.40	200.68	82.45
	Diclofenac	39 598.43	18 550.89	46.85	38 338.77	12 625.82	32.93
Dead	1	276.79	17.97	6.49	191.75	106.17	55.37
	2	127.87	4.16	3.25	73.74	64.44	87.38
	3	**136.08**	132.67	97.49	243.90	212.00	86.92
	Diclofenac	**107 745.64**	84 770.90	78.68	31 769.86	5537.38	17.43

Note: Values in bold show trend exceptions for mean AUC_last_ metabolite peak 3 (136.08) and mean AUC_last_ diclofenac peak (107 745.64); in these birds (which died) the metabolite peak was notably lower and the diclofenac peak notably higher, compared to birds that lived within the test group and both dead and alive birds in the control group.

AUC_last_, Area under the curve to the last measured (quantifiable) time point; s.d., standard deviation; CV, coefficient of variation.

† , All values dose corrected to 1 mg/kg because of the scale of doses used in the study.

### Uric acid analysis

There were no significant differences between the treatment groups when comparing the mean AUC_last_ values or baseline corrections over the scheduled sampling points of 0.25 h, 0.5 h, 1.0 h and 2.0 h post-dosing. All birds (test and control) showed a relatively uniform uric acid concentration at 0 h of sampling, before exposure to diclofenac, and within the reference range as described by Ross et al. (1978) and Wilson and Miles (1988). Because of ethical limitations regarding the volume of blood that could be drawn per bird, it was not possible to evaluate uric acid trends further than 2 h post-dosing.

## Discussion

Diclofenac has been responsible for the mass mortality of three species of Asian vultures. Despite the causality being well established, much still needs to be understood about the mechanism of toxicity and why *Gyps* vultures were so susceptible. At present there is a fair body of literature that suggests a pharmacokinetic and metabolic reason as the underlying driver. Pharmacokinetic data from studies involving diclofenac administration in various other species of birds (Hassan et al. 2018; Naidoo et al. 2007, 2011; Rattner et al. 2008; Swan et al. 2006), suggest an association between mortality and PK parameters that reflect the length of exposure: T_1/2_ and MRT. In most cases, birds with longer T_1/2_ and MRT values died compared to birds that had shorter values. This correlation is most striking in *Gyps* vulture species studied, where the T_1/2_ values are 12.24 h and 16.78 h and the MRT values are 15.11 h and 26.10 h for the Cape Griffon Vulture (CGV) and the African White-backed Vulture (AWBV), respectively (Naidoo et al. 2007). These values are despite a dose equal to that given to the chickens in the study by Naidoo et al. (2007) and much lower than those given to most of the other bird species studied. Other notable differences in their pharmacokinetics are in the AUC_last_ values, which are much higher, and the Cl values, which are much lower than in other bird species. The PK picture is suggestive of zero-order kinetic metabolism in these *Gyps* vultures, or saturation of the intrinsic enzyme metabolising capability.

The specific phase 1 CYP enzyme that metabolises the majority of diclofenac in the human liver is CYP2C9, producing the major and minor metabolites 4′- and 3′-hydroxydiclofenac, respectively (Bort et al. 1999; Leemann et al. 1993). A further minor 5′-hydroxylation metabolite is produced through additional CYP enzymes, including CYP3A4, CYP2C8, CYP2C18 and CYP2C19 (Bort et al. 1999; Shen et al. 1999). From the evidence available, it is reasonable to conclude that metabolism in other mammals is also because of the activity of a member of the CYP2C subfamily. For instance, in the rat and dog, the CYP2C9 homologous enzymes are CYP2C21 and CYP2B11 respectively (Shen et al. 1997; Shou et al. 2003). In the rat, the same metabolites are produced as in humans, that is, 3′-, 4′- and 5′-hydroxydiclofenac (Shen et al. 1997).

Although much less is known about avian CYP enzymes and their role in diclofenac metabolism (Hunter, Mahmood & Martinez 2008), the domestic chicken remains the best-studied species. It is now known that, as in humans, the CYP2 family is predominant in the chicken, and the CYP2C homologs are currently known as CYP2C8/9, CYP2C18, CYP2C23a, CYP2C23b and CYP2C45 (Kawalek et al. 2006; Shang, Jiang & Deng 2013; Watanabe et al. 2013). Furthermore, the CYP2C8/9 homolog has been shown to produce 3′-, 4′- and 5′-hydroxydiclofenac in broiler chickens (Joseph et al. 2006).

By inhibiting CYP2C9 homologs in a test group of domestic chickens (a species where the enzyme is known to function in diclofenac metabolism) with an azole inhibitor, we compared the PK parameters obtained with those from control birds which received only diclofenac. This was in an attempt to ascertain if the length of exposure and related toxicity in vultures may be because of CYP2C subfamily metabolic deficiency. Since the drug is a phase 1 inhibitor, it was expected that it would result in an increase in exposure to diclofenac and reduction in exposure to metabolites. The chicken was specifically chosen since it has a defined LD_50_ of 10 mg/kg (Naidoo et al. 2007), which whilst lower than other bird species, is still higher than for old-world vultures.

For this study, two groups of birds were treated at the range of doses required to establish the LD_50_. The study was able to confirm that the LD_50_ was in the region of 10 mg/kg as previously found in other studies. More so, deaths were confirmed to once again occur with a consistent clinical and pathological picture (Hassan et al. 2018; Naidoo et al. 2007; Oaks & Watson 2011; Swan et al. 2006). An unexpected finding was the death of the birds within 15 min in both groups. With the absence of pathology, the cause of death is possibly attributed to normal cardiac electrical disturbance, such as atrial fibrillation or flutter; this has been documented in humans, even at low doses of diclofenac (Schmidt, Sørensen & Pedersen 2018). All other birds that succumbed died within an expected timeframe of 48 h after dosing, with the exception of a bird from the negative control group, which was euthanised for humane reasons at 56 h post-dosing. Gross pathological signs were also typical, being serosal surface deposition of uric acid crystals and varying degrees of nephrosis.

The original aim of the study was to determine if a CYP inhibitor would make diclofenac more lethal. From the results, it was evident that this was not the case and is likely an indication of insufficient inhibition of the enzyme, either from too low a dose or too short a duration. For the chosen dose, this was the listed dose for birds and was a convenient dose to administer intraperitoneally. The absence of a statistical difference between treatment groups, either for the PK parameters or for comparison of the diclofenac:metabolite ratio peaks, may also be as a result of sample size used – only eight birds from the test group and nine from the control group. We also acknowledge a shortcoming in the study design in that different individuals were used between treatment groups, and therefore inter-subject variation is a complicating factor; that is, as this was an LD_50_ toxicity study, it was not possible to use a crossover study design.

Evident from the study is the large coefficient of variation in the obtained pharmacokinetic parameters, which is indicative of a large intrasubject variability. This is not surprising as the CYP activity of various individuals is well known to vary, with some individuals being rapid metabolisers and others slow metabolisers (Brunton et al. 2018; O’Brien, Chan & Silber 2004; Paulson et al. 1999). Thus, a more valid indicator of effect in this study is in the PK trends and in the difference between the alive and dead animal, both within and between groups. Despite a failure to indicate statistical significance, a trend was starting to emerge to suggest that some CYP inhibition resulted. The test group of chickens which received a CYP inhibitor demonstrated a mean PK curve suggestive of metabolic inhibition when compared to the control group, evident in the geometric mean values for AUC_last_ (0.5 μg/mL*h vs 0.4 μg/mL*h), C_max_ (0.61 μg/mL vs 0.41 μg/mL) and clearance (1.52 L/h*kg vs 1.59 L/h*kg). Birds with suppressed metabolism would be expected to have a higher AUC_last_. Clearance (based on AUC_last_) can be expressed per clearing organ through the following equation:

Cl=Q×E, [Eqn 8]

where Q is the blood flow to the organ and E represents the ‘extraction ratio’ or percentage of drug removed from the blood by that organ during a single passage (Toutain & Bousquet-Mélou 2004). As it was not expected that changes in blood flow to metabolising organs would impact clearance in this study (Naidoo & Swan 2009), clearance would be related to the extraction ratio, E. With this being dependent on the intrinsic metabolising capability of an organ system, the lower geometric mean clearance for the CYP-inhibited test group of birds suggests capacity limited metabolism as a function of CYP enzyme inhibition as a plausible cause in support of the AUC_last_ values seen.

When the within-groups effects are compared, the PK differences become more pronounced. In dead test group birds, there was higher exposure to diclofenac compared to dead control group birds (Table 1), as evidenced by the higher geomean AUC_last_ (0.87 μg/mL*h vs. 0.31 μg/mL*h) and C_max_ values (1.24 μg/mL vs. 0.24 μg/mL) and lower Cl values (0.88 L/h*kg vs. 0.32 L/h*kg). The presence of data from the two highest-dosed birds (both died within 15 min of dosing) in the test group may have increased the reflected difference in exposure between groups further and may be a factor in why T_1/2_ and MRT values are higher in dead control birds (the two highest-dosed birds contributed to the higher values for these parameters), where one would have expected them to be higher in test birds.

From a metabolite aspect, the dead birds had lower ratios of metabolite to diclofenac than those that survived. This would support the assertion that CYP enzymes are involved with metabolism and that lower activity will result in death. Whilst not the intention of the study, this confirmed earlier findings that toxicity is driven by metabolic capacity. An unexpected finding from this study was seen when comparing the metabolites between the two groups. One would have expected the CYP enzyme inhibition (test) group to have lower metabolites. This was not the case, and the opposite was evident. As mentioned above, this once again indicates that CYP inhibition did not occur significantly. More so, it shows how variable the CYP enzyme is in a species. This result may also explain why the chicken is more resilient than the vulture; that is, the higher degree of natural variation in CYP metabolism would explain the higher LD_50_ in the chicken in comparison to the vulture.

## Conclusion

Whilst there was no significant difference in the LD_50_ for chickens given a known CYP2C9 inhibitor and those not, the mean PK curve and the ratio of HPLC diclofenac to metabolite peaks for the dead birds dosed with the inhibitor were suggestive of partial inhibition of CYP2C functioning playing a role in toxicity. To confirm this, a larger sample size would be needed to cater for the large intrasubject variability seen.
